# Lifestyles, Self-Esteem and Mental Well-Being in Students Transitioning to Higher Education

**DOI:** 10.3390/healthcare14060799

**Published:** 2026-03-21

**Authors:** Luís Loureiro, Armando Silva, Ana Teresa Pedreiro

**Affiliations:** 1 Health Sciences Research Unit, Nursing School, the University of Coimbra, Avenida Bissaya Barreto, 3004-011 Coimbra, Portugal; 2 School of Economics, Management and Political Science, University of Minho, Campus de Gualtar, Building 8, 4710-057 Braga, Portugal

**Keywords:** lifestyles, self-esteem, mental well-being, higher education

## Abstract

Introduction: Lifestyle is characterized by identifiable behavioral patterns that can affect individuals’ health, and is considered one of the predominant factors for maintaining both quality of life and people’s health. This triad (lifestyle, quality of life and health) is closely associated with well-being. Objective: The aim of this study is to evaluate the relationship between lifestyles, well-being, and self-esteem in students who have completed secondary education and are in the process of transitioning to higher education. Methods: Data were collected using a questionnaire of sociodemographic (e.g., age, gender) and physical (e.g., BMI) variables, a Self-Esteem Scale, a Well-Being scale, and the FANTASTICO Lifestyle questionnaire. Statistical analysis was performed using canonical correlation analysis and a Structural Equation Model. Results: The sample consisted of 235 students, with a mean age of 18.4 years. Canonical correlation analysis revealed that lifestyle explains 58.5% of the variance in mental health. The first (most important) canonical function (r = 0.86; *p* < 0.001) highlighted that the domains of introspection, sleep/stress management, and family/social support are the primary predictors of higher levels of self-esteem and psychological well-being. Structural Equation Modeling confirmed that lifestyle positively predicts psychological well-being through both direct and indirect pathways (β = 0.172; 95.0% BC CI [0.095, 0.253]). Self-esteem emerged as a significant partial mediator in this relationship, suggesting that healthy lifestyle habits reinforce the perception of personal competence, which, in turn, enhances emotional adjustment. Together, these findings validate the dynamic triad between behavior, self-perception, and well-being during the transition to higher education. Conclusions: This study shows that the transition to higher education is a pivotal period where lifestyle patterns significantly shape students’ psychological adjustment. The findings confirm that a healthy lifestyle, specifically centered on stress management, sleep, and social support, serves as a robust predictor of both self-esteem and psychological well-being. By identifying self-esteem as a key partial mediator, the results suggest that healthy habits do more than just improve physical health.

## 1. Introduction

The transition to higher education (HE) is a critical period marked by personal, social, and academic challenges that have a profound impact on young students’ mental health (MH) and well-being (WB). For a substantial proportion of young people, this transitional period is accompanied by separation from family and close social relationships, together with a sudden increase in autonomy and responsibilities [1,2,3,4,5,6,7].

Evidence shows that many young people do not feel prepared to cope with these new demands, experiencing high levels of stress and anxiety during the transition and adaptation process to HE. Many of these difficulties may persist throughout their studies, contributing, for example, to the emergence of MH problems [8,9,10]. The evidence also points to an increased vulnerability to problem behaviors, particularly during this transitional phase. In this context, the relationship between lifestyles (LS) and MH is highlighted, with healthy lifestyles, high self-esteem, and social support being considered key determinants of MH and WB among HE students [11,12,13]. It is therefore repeatedly stated that a successful transition and integration constitute essential conditions for academic success in all its dimensions. In addition to this substantial body of evidence, studies indicate that behaviors such as regular physical activity, adequate sleep, balanced nutrition, and social engagement—components of lifestyle—are associated with a reduction in symptoms of anxiety, depression, and stress [14,15,16,17,18].

Regarding the relationship between the variables that may contribute to understanding this phenomenon, self-esteem is often identified as a mediator between well-being (WB) literacy and resilience, promoting greater life satisfaction and adaptation to the new university environment and context [4,5]. In addition, social support emerges as a protective factor that enables young people to cope with everyday challenges [7], whereas substance use, including alcohol and tobacco consumption, physical inactivity, and unbalanced nutrition—associated with low self-esteem—increase young people’s vulnerability and may compromise their developmental potential [6,9,12,19].

The relationship between lifestyle (LS), self-esteem, and WB can be understood as a dynamic and interdependent triad, in which each element influences and is influenced by the others.

Lifestyle (LS), understood as patterns of behavior related to physical activity, nutrition, substance use, sleep, stress management, and social relationships, constitutes central determinants of health. When these behaviors are health-promoting, they tend to reinforce the perception of personal competence and control over one’s life, aspects that are closely linked to self-esteem. In turn, positive self-esteem fosters healthier choices, creating a virtuous cycle between behavior and the perception of personal worth.

Individuals with higher levels of self-esteem demonstrate greater self-regulation capacity, increased resistance to social pressure, and a stronger predisposition to invest in healthy practices. Conversely, a LS characterized by physical inactivity, inadequate nutrition, or excessive alcohol consumption may be associated with feelings of body dissatisfaction, guilt, or failure, thereby weakening self-esteem. This fragility may negatively affect psychological well-being (PWB), manifesting through anxiety, demotivation, or lower life satisfaction.

Evidence shows that healthy habits (adequate sleep, balanced nutrition, regular physical activity) are associated with a lower prevalence of depressive and anxious symptoms among university students [1,3,18]. High self-esteem acts as a protective factor against psychological distress and is positively related to the adoption of these healthy behaviors. Overall, studies demonstrate robust associations between healthy habits, high self-esteem, and PWB [1,2,6].

A healthy LS contributes to greater energy, better emotional regulation, and more satisfying interpersonal relationships, thereby enhancing overall WB. Simultaneously, higher levels of WB reinforce the motivation to maintain healthy habits and consolidate positive self-esteem. Thus, the triad of LS, self-esteem, and WB should be understood as a cumulative circular system, in which interventions in one dimension may generate significant effects on the others, particularly in educational and health promotion contexts.

While the connection between lifestyle and well-being is well documented, the current research often focuses on these factors separately, overlooking the intricate and non-linear relationships between various lifestyle aspects and mental health outcomes. Additionally, there is a notable lack of research on specific factors, such as self-esteem, that play a role in these connections as students transition to higher education, especially among nursing students.

**Figure 1:**
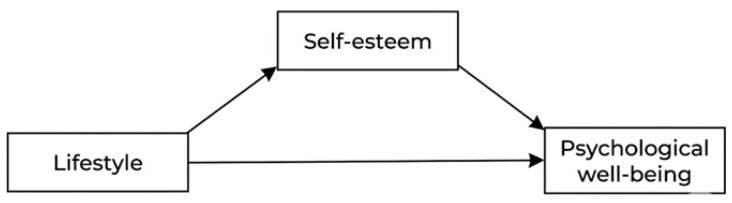
Theoretical path model of the mediating effect of self-esteem on the relationship between lifestyle and psychological well-being.

Based on the literature review, we propose a theoretical model (Figure 1) that explores the dynamic interplay between these variables, hypothesizing that self-esteem acts as a critical mediator between healthy behaviors and overall psychological well-being during the academic transition.

In this context, our objectives are to (a) assess the interrelationships between lifestyle (LS), psychological well-being (PWB), and self-esteem among students transitioning to higher education; and (b) examine the mediating role of self-esteem, quantifying the direct and indirect pathways through which healthy behaviors influence mental health (MH).

## 2. Materials and Methods

### 2.1. Research Design and Hypotheses

This study used a descriptive correlational design. Within this framework, the following research hypotheses were formulated:

**H1.**  *The lifestyle (LS) profile of young students transitioning to higher education (HE) is associated with their mental health (MH) profile, as expressed by self-esteem and psychological well-being (PWB);*

**H2.**  *Healthier LS predict higher levels of PWB among young students transitioning to HE, both directly and indirectly, through the positive mediating effect of self-esteem.*

### 2.2. Settings and Data Collection

A quantitative, cross-sectional, and descriptive correlational study was conducted in September 2024, during student orientation week. Data were collected at a large nursing school within a university located in the central region of mainland Portugal. The questionnaire was administered in the classroom using the Google Forms platform, with the researchers present at each administration session. Access to the questionnaire was provided via a QR code in the classrooms. Participants provided electronic informed consent, confirming that they had understood the study objectives and agreed to participate voluntarily. The average time for completing the questionnaire was 11.45 min.

### 2.3. Data Collection Instruments: Sample Characterization Questionnaire

Part 1 of the questionnaire included several items aimed at characterising the sample and describing students’ individual and contextual profiles. This section comprised personal data (age and gender), parents’ educational qualifications, and indicators of academic trajectory (degree programme as first choice and possible reconciliation of study and work).

In parallel, the questionnaire included socioeconomic and behavioral questions, namely household composition and income, status as a displaced student and living in shared accommodation, involvement in volunteer activities, and indicators related to leisure habits and financial expenditure associated with recreational activities. Anthropometric variables (weight and height) were also collected, allowing for the calculation of body mass index as an indicator of nutritional status.

#### 2.3.1. FANTASTIC Lifestyle Questionnaire

The FANTASTIC Lifestyle Questionnaire [20] is a multidimensional assessment instrument designed to evaluate health-related behavioral patterns, integrating different domains of lifestyle (LS) that influence physical, psychological, and social well-being.

The questionnaire includes a total of 30 items and explores ten domains of the physical, psychological, and social components of lifestyle, represented by the acronym “FANTASTICO,” where F—family and friends; A—physical activity/associations; N—nutrition; T—tobacco; A—alcohol and other drugs; S—sleep/stress; T—work/personality type; I—introspection; C—health and sexual behaviors; and O—other behaviors. The items have three response options, represented by numerical values of 0, 1, or 2.

Its design is based on the premise that LS results from the dynamic interaction between individual, social, and environmental factors, constituting an important determinant of health throughout the life course [21].

From a psychometric perspective, the FANTASTIC questionnaire has demonstrated adequate validity and reliability in different cultural contexts and is widely used in studies with young populations. In this study, Cronbach’s α was 0.87.

#### 2.3.2. Self-Esteem Scale (SES)

In this study, the Portuguese version [22] of the SES [23] was used. This is a widely employed instrument for assessing global self-esteem, understood as the individual’s subjective evaluation of their own worth and competence. The scale consists of 10 self-report items, formulated as positive and negative statements, rated on a four-point Likert scale ranging from strongly disagree to strongly agree.

The total score is obtained by summing the items, after reversing the negatively worded items, with higher values indicating higher levels of self-esteem. Its unidimensional structure, combined with adequate validity and reliability indicators across different cultural contexts, has supported its widespread use in scientific research with adolescent and adult populations. In this study, the internal consistency was Cronbach’s α = 0.90.

#### 2.3.3. Psychological General Well-Being Index (PGWB-S)

The PGWB-S [24], in its short version and adapted for the Portuguese population [25,26], conceptualizes well-being as a construct reflecting positive emotional and affective states, as well as the absence of anxious and depressive symptomatology.

The PGWB-S assesses an individual’s subjective perception of their mental health through six items covering dimensions such as anxiety, vitality, emotional control, and happiness. The response format is based on a six-point Likert scale (ranging from 0 to 5), where respondents indicate the frequency or intensity of each state experienced over the past month. The overall score is obtained by summing the items (after reversing negatively worded items), resulting in a total index ranging from 0 to 30, with higher scores indicating higher levels of psychological well-being. In this study, the reliability value was Cronbach’s α = 0.82.

### 2.4. Data Analysis

In this study, data analysis was performed using IBM SPSS Statistics (Version 30) and AMOS Version 30. Appropriate summary statistics (means and standard deviations) and absolute and percentage frequencies were calculated. Canonical correlation analysis (CCA) was employed. This multivariate statistical technique allows for the examination of interrelationships between sets of variables. The application of this model was preceded by verification of its fundamental assumptions, namely the multivariate normality of the variables, the linearity of the relationships between pairs of variables from both sets, and the absence of severe multicollinearity. Additionally, the homogeneity of variance–covariance matrices and the adequacy of the sample size were ensured [27].

Mediation analysis was conducted using Structural Equation Modeling (SEM) in AMOS, with prior verification of multivariate normality assumptions. To test the significance of the indirect effect, the maximum likelihood procedure was applied in conjunction with the bootstrapping technique (with 2000 resamples and a 95% confidence interval). Mediation was confirmed by the statistical significance of the indirect effect (*p* < 0.05) and the absence of zero in the bias-corrected confidence interval [28].

The combined use of these two types of analyses—namely CCA and mediation analysis via SEM—is methodologically justified as they address complementary objectives. CCA is appropriate for initially examining, in an exploratory and multivariate manner, the overall relationship between the two sets of variables. Based on the evidence generated and considering the theoretical derivation of the constructs, mediation analysis via SEM then allows for hypothesis-driven, confirmatory testing, quantifying direct and indirect effects for the mediated component.

### 2.5. Ethical Considerations

Both the study and the questionnaire were approved by the institution’s administration and the Ethics Committee of the Health Sciences Research Unit: Nursing (P147032013). The inclusion criteria were provided to allow individuals to voluntarily agree to participate in the study and to provide electronic consent, when required.

## 3. Results

The study sample consists of 235 young university students, with a mean age of 18.43 years (±0.83). Regarding gender distribution, there is a marked predominance of females, representing 85.53% of the sample, compared to 14.47% males. Concerning academic and professional background, the majority of participants are exclusively dedicated to their studies (92.34%), while only 7.66% combine academic activity with work. Only 23.40% of students report being able to enroll in their first-choice program, suggesting that a large majority (76.60%) may be attending an alternative course to their initial preferences.

Regarding the socio-familial and residential context, 74.89% of students are living away from their usual place of residence, in contrast with 25.11% who remain with their family household. Concerning parental educational attainment, 36.17% of mothers and 25.96% of fathers have completed secondary education, with higher education being more prevalent among mothers (28.09% with a bachelor’s degree) than fathers (16.60% with a bachelor’s degree). Finally, in the scope of social participation and active citizenship, the majority of the sample do not engage in volunteer activities (85.53%), with only 14.47% of the surveyed youths participating regularly.

Considering anthropometric indicators, the nutritional profile of the sample was determined through body mass index (BMI). In the BMI results by category, most participants are within the normal weight range (74.89%), followed by 13.19% who are overweight and 11.06% who are underweight. Grade I obesity is residual, accounting for only 0.86%.

Regarding the summary statistics of the scales (Table 1), the dimensions of FANTASTICO generally exhibit high mean scores relative to their respective ranges, suggesting an overall favorable profile across the different domains of lifestyle. Other behaviors (O) (mean = 3.79; SD = 0.60) and introspection (I) (mean = 3.70; SD = 1.43) stand out as the dimensions with the highest mean values, whereas work/personality type (T) (mean = 3.21; SD = 1.19) and sleep/stress (S) (mean = 3.26; SD = 1.43) show slightly lower means. Relative dispersion, measured by the coefficient of variation (CV), indicates greater heterogeneity in sleep/stress (43.87%) and physical activity/associations (40.45%), while alcohol and other drugs (9.67%) and the total FANTASTICO score (13.27%) reveal higher consistency among participants. Overall, the FANTASTICO score presents a mean = 85.06 (SD = 11.29), reflecting relatively low variability for the observed average level.

Regarding distribution shapes, there is predominantly negative skewness (e.g., between −0.06 and −3.30), consistent with a higher concentration of responses at elevated values across several dimensions. This pattern is particularly pronounced in other behaviors (skew = −3.30), alcohol and other drugs (skew = −2.15), tobacco (skew = −1.70), and family and friends (skew = −1.33). Kurtosis is close to zero in most dimensions (e.g., S = −0.44; T = −0.26; I = −0.35), suggesting approximately mesokurtic/slightly platykurtic distributions; however, higher values occur in other behaviors (kurtosis = 12.04) and alcohol and other drugs (kurtosis = 6.87), indicating more “peaked” and concentrated distributions.

Regarding the Self-Esteem Scale (SES), it presents a mean = 28.64 (SD = 4.82; CV = 16.83%), with an almost symmetric distribution (skew = 0.00) and moderate positive kurtosis (0.60), whereas the Psychological Well-Being Scale (PGWB-S) shows a mean = 16.68 (SD = 4.73; CV = 28.36%), with slight negative skewness (skew = −0.26) and kurtosis close to zero (−0.06), suggesting greater heterogeneity in well-being scores compared to self-esteem.

In the next step, canonical correlation analysis (CCA) was performed. In the first dataset, the FANTASTICO dimensions were included, while in the second dataset, SES and PWB were incorporated. The analysis (Table 2) revealed that the first canonical function is statistically significant and exhibits a very strong correlation (*r* = 0.868; WL = 0.216; *p* < 0.001). Therefore, a strong multivariate relationship can be observed between the pattern displayed by the FANTASTICO dimensions and self-esteem and PWB. Regarding the second function, although it is statistically significant (*p* < 0.001), the correlation is very modest, and its explanatory power is negligible (r = 0.355; WL = 0.874; *p* < 0.001).

Table 3 presents the canonical loadings and the cross-loadings (indicated in parentheses). The cross-loadings were prioritized for interpreting the relationship between the variable sets, as simple canonical loadings can sometimes be inflated by shared variance within their own set. Therefore, cross-loadings provide a more conservative and accurate measure between the lifestyle dimensions and the mental health constructs.

Regarding both canonical loadings and cross-loadings, (Table 3), it can be observed that in the first function, the canonical loadings in Set 2 are very high and negative (PGWB-S = −0.950 and SES = −0.815). These data indicate that this function essentially reflects a factor of psychological adjustment and functioning, with greater weight on well-being (PWB). In the same function, within Set 1, the most relevant canonical loadings are introspection (I) (−0.874), followed by sleep/stress (S) (−0.774), work/personality type (T_A) (−0.733), and family and friends (F) (−0.554). This suggests that higher scores in these dimensions (I, S, T_A, F) tend to be associated with higher self-esteem and better psychological well-being, implying that balance in these specific lifestyle areas is a key “predictor” of university students’ psychological health, explaining the shared variance between the two datasets.

Regarding the second function, Set 2 separates self-esteem (−0.580) and well-being (0.313), indicating a specific contrast (with opposite signs). In Set 1, the loadings with the highest values are C (health and sexual behaviors) (−0.574) and A_A (alcohol and other drugs) (0.445). Although statistically significant, as previously noted, the second function has limited practical utility.

The results of the proportion of explained variance (Table 4) show that Canonical Function 1 best summarizes the relationship between the IVs and MH-PWB. On the IV side, this function explains, on average, 25.3% of the variance of the dimensions (IV 1 by IV = 0.253), indicating that it captures a relevant—although not complete—portion of the IV construct. On the MH-PWB side, the same function represents the set very well (MH-PWB by Self = 0.780), suggesting that self-esteem and PWB are strongly aligned in this linear combination. In terms of redundancy, Function 1 shows a substantive association: IVs explain 58.8% of the variance in MH-PWB (MH-PWB by IV = 0.588), whereas MH-PWB explains 19.9% of the variance in IVs (IV by MH-PWB = 0.190), reinforcing the idea that lifestyle patterns have a high capacity to capture variability in mental health status, more so than the reverse.

Canonical Function 2 has a clearly more limited contribution. Although it still explains some variance within each set (IV: 9.4%; MH-PWB: 22.0%), the capacity of one set to explain the other is very low, with redundancies of only 1.2% (IV by MH-PWB) and 2.8% (MH-PWB by IV). Thus, this second function appears to primarily reflect a residual/specific pattern that adds little to the understanding of the overall relationship between IVs and MH.

**Figure 2:**
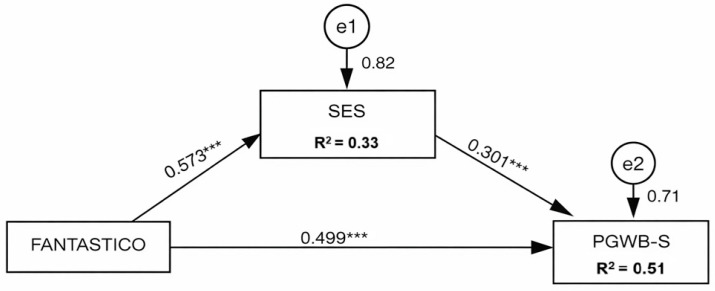
Standardized theoretical path model of the mediating effect of SES on the relationship between EV and BEP. *** = *p* < 0.001.

When investigating the mediation effect, we constructed a model (Figure 2) in which the effect of IVs (global score of FANTASTICO) on PWB (PGWB-S) was tested, mediated by self-esteem (SES).

The mediation model indicated that IVs are significantly associated (*p* < 0.001) with both self-esteem and PWB. In particular, IVs positively predicted self-esteem (β = 0.573; *p* < 0.001), and self-esteem positively predicted PWB (β = 0.301; *p* < 0.001). Additionally, a significant direct effect of IVs on PWB remained after including the mediator variable (β = 0.499; *p* < 0.001), suggesting that the relationship between IVs and PWB is not fully explained by self-esteem.

Analysis of the indirect effects supported partial mediation by self-esteem. The indirect effect of FANTASTICO on PGWB-S via SES was statistically significant (β = 0.073), with a 95% bias-corrected bootstrap confidence interval ([0.039; 0.109]); in standardized terms, the indirect effect was β = 0.172, 95% CI: bias-corrected ([0.095; 0.253]). Overall, these results indicate that higher IVs are associated with greater psychological well-being, in part through higher self-esteem, while a robust direct component between IVs and PWB remains.

## 4. Discussion

The present study aimed to analyze, in young students transitioning to higher education (nursing program), the relationship between lifestyle behaviors (IVs), self-esteem, and psychological well-being (PWB) during a period recognized as critical for these young people [1,2,3,4,5,6,7].

An initial finding is that the results support the formulated hypotheses. Overall, the CCA and the mediation model converge on a key point: during the transition to higher education, it is not merely the IVs that are associated with mental health, but rather a specific pattern of related dimensions—such as self-regulation, sleep/stress, academic/occupational functioning, and socio-affective support—that is strongly linked to a positive psychological functioning axis (self-esteem and PWB).

### 4.1. Sample Profile and Transition Context

The contextual characterization of the sample reinforces the relevance of what was mentioned in the introduction. The high proportion of students living away from home (74.9%) is consistent with the notion of familial separation and a relative loss of daily routines and close social support, factors frequently identified as contributors to increased perceived stress and emotional vulnerability [1,2,3,4,5,6,7].

Furthermore, the fact that only 23.4% of students are enrolled in their first-choice program may constitute a factor affecting motivation and a sense of vocational coherence, potentially leading, in the short and medium term, to frustration and uncertainty—dimensions often associated with stress and internalizing symptoms during the adaptation phase to higher education [8,9,10]. Nonetheless, the mental health and lifestyle profile of these youths, based on the scale statistics, suggests generally favorable lifestyle patterns and relatively high levels of self-esteem and psychological well-being.

While these results indicate good overall mental health, they do not preclude the existence of vulnerable subgroups. The observed variability in factors such as sleep/stress and physical activity suggests heterogeneity, which may impact how students manage daily life during this phase. The evidence also shows considerable interindividual variation: while some students stabilize or improve, others experience disruptions in routines, associated with higher anxiety and lower well-being [8,9,10].

Negative skewness in several FANTASTICO dimensions (notably “other behaviors” and “alcohol and other drugs”) suggests a concentration of responses at higher levels, consistent with rather positive perceptions in these domains.

### 4.2. Relationship Between Lifestyle and Mental Health

The CCA showed that the first canonical function exhibits a very strong correlation (r = 0.864) and high redundancy of lifestyle on mental health (0.585), meaning, substantively, that a specific pattern of lifestyle dimensions captures a substantial portion of the shared variability of self-esteem and psychological well-being. This evidence aligns with the literature, which posits that healthy lifestyles function as fundamental determinants of mental functioning in young students [11,12,13], as well as studies linking physical activity, adequate sleep, balanced nutrition, and social engagement to lower anxiety symptoms and better emotional regulation [14,15,16,17,18].

The loadings of Function 1 indicate that not all dimensions contribute equally. “Introspection,” “sleep/stress,” “work/personality type,” and “family and friends” were the domains most aligned with the “psychological axis” (self-esteem + psychological well-being). This finding is theoretically justified and relevant in the transition to higher education.

Firstly, the sleep/stress factor is directly related to changes in schedules, study load, new demands for autonomy/responsibility, and anticipatory anxiety frequently described during this phase of higher education integration [1,2,3,4,5,6,7,8,9,10].

Secondly, work/personality type and introspection capture characteristics related to self-regulation, organization, and coping—skills that help manage and deal, for example, with academic pressure in daily life.

Thirdly, family and friends indicates that relational dimensions remain a structuring element, even when students are living away from family [7], potentially moderating stress reactivity and supporting healthy routines.

In practical terms, the hierarchy discussed here suggests that interventions focusing solely on positive health behaviors, such as physical exercise and healthy nutrition, are insufficient if they do not address emotional management, academic integration, and social support. During this phase, the data indicate that dimensions related to stress/sleep, self-regulation, and social support appear to be the most immediate structures connecting lifestyle and mental health.

### 4.3. Mediation Effect of Self-Esteem

The mediation model demonstrated a structure consistent with the dynamic triad presented in the introduction: lifestyle behaviors (LS) predict self-esteem (β = 0.573), self-esteem predicts well-being (β = 0.301), and LS maintain a direct effect on well-being even after inclusion of the mediator (β = 0.499). The indirect effect was supported by 95% bias-corrected confidence intervals (BC CI) (not including zero) (b = 0.073, 95% BC CI [0.039; 0.109]; β = 0.172, 95% BC CI [0.095; 0.253]), supporting the reference made and indicating partial mediation.

These results are consistent with the referenced literature, which positions self-esteem as a resource or protective factor and often a mediator in adaptation processes [4,5]. Conceptually, better lifestyle behaviors can lead to increased self-esteem through several pathways: reinforcement of self-efficacy, greater perceived control, improved body image, and higher engagement in social participation activities.

Higher self-esteem may also contribute to well-being through greater frustration tolerance, increased social initiative, and improved coping strategies—mechanisms relevant when young people face new challenges that may be ambiguous or even threatening [1,2,3,4,5,6,7].

At the same time, the fact that the effect of lifestyle behaviors remains strong suggests that other factors influence well-being without necessarily operating through self-esteem. These include good sleep hygiene and regular physical activity, which promote better emotional regulation and daily stress management, constituting a key contribution to enhanced quality of life and well-being [14,15,16,17,18].

It is also noteworthy that the results show convergence between the two statistical techniques used: CCA, an exploratory multivariate method, and mediation analysis. The former demonstrates that mental health (in this case, psychological well-being) is organized around psychological functioning, being associated with reduced stress, quality sleep, greater introspection, and academic functioning. Meanwhile, the mediation model shows that the association between lifestyle behaviors and psychological well-being can be explained by a psychological resource—self-esteem.

These results fit within the initial premise [11,12,13] that good social support, high self-esteem, and healthy lifestyle routines are fundamental for a successful transition and integration into higher education, with self-esteem also functioning as a mediator in adaptive processes [4,5].

## 5. Conclusions

The present study contributes to understanding the mechanisms that support the mental health of nursing students during the critical transition to higher education.

The results indicate that psychological well-being does not depend solely on individual choices or personal characteristics of the students, but rather reflects an integrated pattern of lifestyle behaviors (IVs) and the conditions they encounter within the higher education institution they attend. It is noteworthy that self-esteem exerts a partial mediation in the relationship between lifestyle behaviors and well-being. These findings suggest that interventions should target the strengthening of psychological resources, as well as institutional academic management policies, guiding their actions in alignment with the institution’s social mission, which includes consideration of the factors influencing students’ well-being.

In sum, the transition to higher education should be understood as a dynamic process that involves both the individual resilience of students and institutional responsibility.

### 5.1. Implications of the Results in the Context of Student Orientation and Integration Practices in Higher Education

The results suggest that strategies for promoting mental health in terms of student orientation and integration are fundamental for personal and academic success throughout higher education. However, these strategies require an integrated approach [1,2,3,4,5,6,7,11,12,13,14,15,16,17,18] that includes, for example, daily stress management programs, mental health first aid initiatives, positive health programs promoting sleep hygiene, self-regulation and planning training, integration groups, and even mentoring, as well as initiatives that foster socio-emotional skills [8,10,29,30,31]. Given that the mediation effect of self-esteem is partial, it is believed that interventions should also act directly on contextual aspects (e.g., schedules, workload, study environments, and institutional support), not solely on psychological domain variables.

### 5.2. Limitations and Suggestions for Future Research

There are several important limitations to consider.

While acknowledging the limitations inherent in this research design, specifically the use of a convenience sample from a single university, several factors bolster the study’s internal and external validity. The sample size (*n* = 253) exceeds the minimum threshold required for SEM, which typically demands at least 10 subjects per parameter to ensure adequate power for multivariate analysis, and the same applies to the CCA. Although a gender imbalance is observed, this reflects the current reality of the nursing profession and its educational context. Although these are nursing students, and in this case, the percentages are close to the reality of the program and profession, which is largely attended and practiced by women. This demographic characteristic may influence the results. In a broader higher education context, samples with more balanced gender distributions should be considered.

An additional limitation is that the questionnaires were administered via self-report. In this sense, social desirability may influence some of the results, which can be particularly relevant for FANTASTICO subscales such as substance use.

It should also be noted that sociodemographic variables (e.g., simultaneous employment; living away from home), clinical variables (e.g., BMI), or factors related to higher education attendance (e.g., whether the program was the student’s first choice) were not included in the analysis. These factors may contribute to explaining the observed relationships and should be incorporated in future studies on this topic.

Finally, we believe that future research should prioritize longitudinal designs that allow for the identification of periods of increased risk and vulnerability, thereby enabling more targeted and robust interventions. This study serves as a robust exploratory analysis within the scope of Portuguese nursing students, as the results provide a foundational benchmark for a conceptual roadmap for future research.

## Figures and Tables

**Table 1:** Descriptive statistics of variables (*N* = 235).

Variables:	Mean	Sd	CV (%)	Skew	Kurtosis
Family and friends—F	3.32	0.99	29.82	−1.33	0.80
Physical activity/associations—A	3.56	1.44	40.45	−0.26	−0.19
Nutrition—N	3.58	1.02	28.49	−0.22	0.53
Tobacco T	3.48	1.01	29.02	−1.70	1.44
Alcohol and other drugs—A	11.06	1.07	9.67	−2.15	6.87
Sleep/stress—S	3.26	1.43	43.87	−0.10	−0.44
Work/personality type—T	3.21	1.19	37.07	−0.07	−0.26
Introspection—I	3.70	1.43	38.65	−0.06	−0.35
Health and sexual behaviors—C	3.58	1.33	37.15	−0.58	0.54
Other behaviors—O	3.79	0.60	15.83	−3.30	12.04
FANTASTICO	85.06	11.29	13.27	−0.38	0.45
SES	28.64	4.82	16.83	0.00	0.60
PGWB-S	16.68	4.73	28.36	−0.26	−0.06

Sd = standard deviation; CV = coefficient variation.

**Table 2:** Canonical correlations.

	Correlation	Eigenvalue	Wilks Statistic	F	Numerator D.F	Denominator D.F.	*p*
1	0.868	3.054	0.216	26.295	20	456	<0.001
2	0.355	0.144	0.874	3.657	9	229	<0.001

**D.F** = degree of freedom.

**Table 3:** Canonical loading and cross loadings (in parentheses) by canonical function and set 1 and 2.

LS	1	2
Family and friends—F	−0.554 (−0.479)	−0.296 (−0.101)
Physical activity/associations—A	−0.350 (−0.303)	0.167 (0.057)
Nutrition—N	−0.205 (−0.177)	−0.194 (−0.066)
Tobacco T	−0.033 (−0.029)	0.268 (0.092)
Alcohol and other drugs—A	0.237 (0.205)	0.445 (0.152)
Sleep/stress—S	−0.774 (−0.669)	0.115 (0.039)
Work/personality type—T	−0.733 (−0.633)	0.331 (0.113)
Introspection—I	−0.874 (−0.755)	−0.076 (−0.026)
Health and sexual behaviors—C	−0.264 (−0.228)	−0.574 (−0.196)
Other behaviors—O	0.049 (0.043)	−0.063 (−0.022)
**MH**		
SES	−0.815 (−0.704)	−0.580 (−0.198)
PGWB-S	−0.950 (−0.821)	0.313 (0.107)

**Table 4:** Proportion of variance explained.

Canonical Variable	EV 1 by EV	EV by MH-PWB	MH-PWB by MH-PWB	MH-PWB by EV
1	0.253	0.190	0.780	0.588
2	0.094	0.012	0.220	0.028

## Data Availability

The data that support the findings of this study are available from the corresponding author, L.L., upon reasonable request because of privacy and ethical restrictions.

## Abbreviations

HE	Higher education
MH	Mental health
WB	Well-being
PWB	Psychological well-being
PGWB-S	Psychological General Well-Being Index
FANTASTICO	FANTASTICO Lifestyle Questionnaire
SES	Self-Esteem Scale
CCA	Canonical correlation analysis
SEM	Structural Equation Model
SD	Standard deviation
CV	Coefficient of variation
MH-PWB	Mental health and psychological well-being
